# SuperAgers, amyloid, and brain resilience: beyond the absence of pathology

**DOI:** 10.1055/s-0046-1817825

**Published:** 2026-03-11

**Authors:** Paulo Caramelli, Wyllians Vendramini Borelli

**Affiliations:** 1 Universidade Federal de Minas Gerais, Faculdade de Medicina, Unidade de Neurologia Cognitiva e do Comportamento, Belo Horizonte MG, Brazil.; 2 Universidade Federal do Rio Grande do Sul, Departamento de Ciências Morfológicas, Porto Alegre RS, Brazil.; 3 Hospital Moinhos de Vento, Centro de Memória, Porto Alegre RS, Brazil.

In the last 15 years there has been an increasing interest in the investigation of oldest-old adults whose memory performance is similar or superior to people aged 20 to 30 years younger. Such individuals with an exceptional cognitive aging profile have been named SuperAgers (SA) 1 or high-performing older adults (HPOA). 2 3 These remarkable cognitive abilities maintained throughout the aging process challenge traditional models that associate aging, cognitive decline, and brain pathology. 4 Research on this topic represents a valuable human model for studying cognitive and brain reserve, as well as brain resilience. Moreover, the field is important in the context of the fast demographic transition experienced by middle-income countries like Brazil, as it can identify the underlying mechanisms of this unusual, albeit positive, outcome, including potential modifiable factors that can be targeted by pharmacological or non-pharmacological interventions.

In this context, the study by Studart-Neto *et al* . 5 published in the current issue of *Arquivos de Neuro-Psiquiatria* represents an important contribution to this debate. The authors used molecular (positron-emission tomography - PET - using 11C-PiB and 18F-FDG tracers) and structural (magnetic resonance) neuroimaging to investigate cortical amyloid deposition, brain glucose metabolism, and gray matter volume in SA. They evaluated 11 SA, 23 age-matched healthy controls (HC80) and 23 healthy controls aged 60 to 69 years (HC60). All three groups were characterized by a high median educational level (16 years).

Four SA (36.4%) were amyloid-positive, a rate comparable to the one observed in the HC80 group (40.9%). Notable, six SA (54.5%) reported subjective cognitive complaints, three of whom were amyloid-positive. Within the SA group, amyloid deposition was significantly associated with episodic memory - specifically, delayed recall scores in the Rey Auditory Verbal Learning Test. The SA group displayed increased metabolism in the left anterior left cingulate gyrus and the left caudate in comparison to the HC80 group. Interestingly, SA also presented areas of increased brain metabolism compared to HC60. The authors conclude that while SA exhibit similar amyloid burden to age-matched individuals and that amyloid deposition impaired their episodic memory performance, the findings of increased metabolism and gray matter volume in areas of the brain salience network and in the striatum may suggest that these regions support successful cognitive aging.

The study of Studart *et al* . 5 reinforces the notion that successful cognitive aging is not defined solely by reduced Alzheimer's disease (AD) pathology, but also - and perhaps primarily - by mechanisms of brain resilience and compensation. Until recently, the scientific literature has been mostly dominated by unimodal imaging approaches, which limit the integration of molecular pathology, brain function, and structure. In this context, the present study advances the field by combining structural magnetic resonance, amyloid- and FDG-PET, and by investigating these biomarkers in relation to subjective complaints and cognitive performance.

A striking study finding is that cortical amyloid burden in SA was comparable to that observed in age-matched controls. Around one-third of SA presented positive amyloid-PET scans, a similar proportion to that reported in studies with large samples of cognitively unimpaired older adults. 6 7 This finding aligns with growing evidence that amyloid deposition in the brain is common in advanced age and, in isolation, insufficient to fully account for cognitive decline. 6 Importantly, however, Studart-Neto *et al* . 5 demonstrate that within the SA group, higher cortical amyloid burden was associated with worse episodic memory performance. Thus, despite preserved overall cognitive performance, amyloid pathology appears to exert a measurable negative effect on episodic memory in these individuals.

Compared with HC80 participants, SA exhibited increased regional brain glucose metabolism in the anterior cingulate cortex and caudate nucleus, as well as increased gray matter volume in the putamen. A previous Brazilian study also found increased metabolic activity in the anterior cingulate cortex in HPOA/SA compared to controls. 8 These regions are key components of frontostriatal circuits and the salience network, which are involved in monitoring, executive control and motivation, and the integration of cognitive and emotional processes. 9 Enhanced activity of these circuits suggests that they may play a critical compensatory role, allowing individuals to maintain high levels of cognitive function in the presence of amyloid pathology. From this perspective, successful cognitive aging may rely less on the integrity of memory-related medial temporal structures and more on the efficiency of higher-order control networks that optimize cognitive processing.

These observations have important conceptual implications. Rather than supporting a model of complete resistance to AD pathology, the findings favor resilience and compensation framework, in which cognitive performance is maintained despite the presence of potentially deleterious brain changes. 10 11 In this regard, the metabolic and structural findings reported in the study are particularly relevant.

Some limitations are acknowledged by the authors. The small sample size, inherent to the paucity of the SA phenotype, limits statistical power and generalizability. The cross-sectional design precludes causal inferences, particularly regarding the longitudinal impact of amyloid burden on cognition in SA. Nevertheless, the methodological rigor and convergence of the findings with the literature strengthen the conclusions.

In summary, the study by Studart-Neto *et al* . 5 contributes to our understanding of exceptional cognitive aging by demonstrating that older adults with exceptional cognitive abilities may harbor amyloid pathology while maintaining cognitive performance through brain features associated with resilience. These findings highlight the importance of moving beyond pathological models and incorporating markers of neural compensation and reserve. Moreover, identification of potentially enhancing mechanisms of the salience network and striatal function may open new avenues for dementia prevention and for promoting successful cognitive aging.
